
JOURNAL INFORMATION
==============================
NLM Title Abbreviation: ScientificWorldJournal
Journal ID: ScientificWorldJournal
NLM Journal ID: 101131163
Journal ID: TSWJ

The Scientific World Journal

ISSN: 2356-6140
EISSN: 1537-744X
Publisher: Wiley

ARTICLE INFORMATION
==============================
PMCID: PMC5763699
PMID: 20623091
DOI: 10.1100/tsw.2010.105
Article ID: 458421
Article version: 1
Subjects: Clinical Images

Osteosclerotic Prostatic Metastasis

Chedgy Edmund C.P. 1 *
Niematallah Ibrahim 2
Hawary Amr 1
1 Department of Urology, Weston General Hospital, Weston-Super-Mare, Somerset, UK
2 Department of Urology, Ysbyty Gwynedd, Penrhosgarnedd, Bangor, Gwynedd, UK
*Edmund C.P. Chedgy: edchedgy@hotmail.comAcademic Editor: Daniel Batlle

Publication date: 2010
Electronic publication date: 2010 Jul 6
Volume: 10
First page: 1330
Last page: 1331
Received 2010 Apr 25; Revised 2010 May 7; Accepted 2010 May 7
Copyright: Copyright © 2010 Edmund C.P. Chedgy et al.
Copyright year: 2010
License: This is an open access article distributed under the Creative Commons Attribution License, which permits unrestricted use, distribution, and reproduction in any medium, provided the original work is properly cited.
License URL: https://creativecommons.org/licenses/by/3.0/

Keywords: prostate, cancer, pelvis, metastasis, osteosclerotic

==============================
Clinical Image
*TheScientificWorldJOURNAL* (2010) 10, 1330–1331
ISSN 1537-744X; DOI 10.1100/tsw.2010.105

TheScientificWorldJOURNAL
www.thescientificworld.com

### Osteosclerotic Prostatic Metastasis

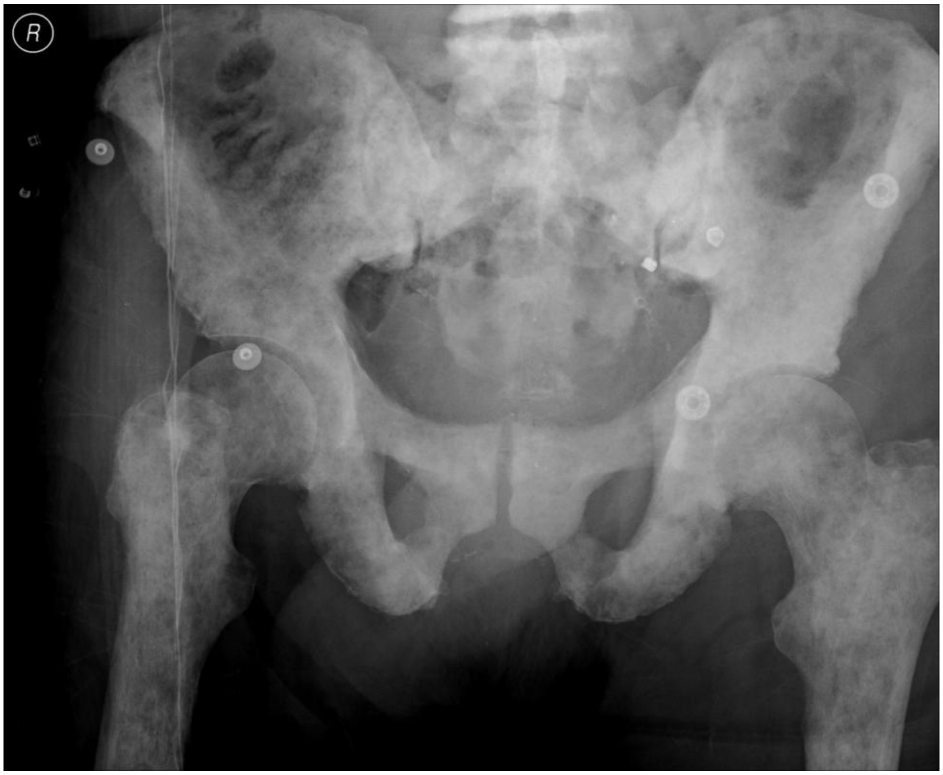

**FIGURE 1.** Anteroposterior radiograph of the pelvis demonstrating multiple osteosclerotic metastases from prostate adenocarcinoma. The sclerotic metastases are seen throughout all the skeletal structures.

Edmund C.P. Chedgy[1,*], Ibrahim Niematallah[2], and Amr Hawary[1]

*[1]Department of Urology, Weston General Hospital, Weston-Super-Mare, Somerset, U.K.; [2]Department of Urology, Ysbyty Gwynedd, Penrhosgarnedd, Bangor, Gwynedd, U.K.*

**E-mail:** edchedgy@hotmail.com

*Received April 25, 2010; Revised May 7, 2010; Accepted May 7, 2010; Published July 6, 2010*

**KEYWORDS**: prostate, cancer, pelvis, metastasis, osteosclerotic

An 80-year-old retired engineer with known prostate cancer was admitted under the care of the physicians generally unwell, complaining of tiredness, with associated agonising bilateral hip pain. On admission,

*Corresponding author.
©2010 with author.
Published by TheScientificWorld; www.thescientificworld.com plain pelvic X-rays revealed multiple osteosclerotic metastases characteristic for prostatic cancer. This radiologic finding was confirmed by the clinical finding of a hard, malignant-feeling prostate gland on digital rectal examination and a prostatic-specific antigen of 290 ng/ml. The patient was started on hormonal treatment, namely, luteinising hormone releasing hormone analogues (LHRH), and referred for palliative radiotherapy for symptomatic control and to the palliative care team for longer-term care plans.

Osteosclerotic metastases from prostate cancer have been described in skeletons as far back as the first century A.D.[1] and more recently the Middle Ages[2]. Patients with only metastases to bone can survive up to 10 years or more. Current treatments of bony metastases are limited to bisphosphonates, with the aim of improving quality of life by reducing the risk of developing pathological fractures, with no survival benefits[3].

Prostate cancer metastases on plain pelvic radiograph are much less frequently encountered in current clinical practice, as it is not considered a part of the workup for prostate cancer patients[4] and is usually only seen as an incidental finding when patients are investigated for bone pains or following falls.

### REFERENCES

1. Grévin, G., Lagier, R., and Baud, C.A. (1997) Metastatic carcinoma of presumed prostatic origin in cremated bones from the first century A.D. *Virchows Arch.* **431(3),** 211–214.
2. Tkocz, I. and Bierring, F. (1984) A medieval case of metastasizing carcinoma with multiple osteosclerotic bone lesions. *Am. J. Phys. Anthropol.* **65(4),** 373–380.
3. Clezardin, P. and Teti, A. (2007) Bone metastasis: pathogenesis and therapeutic implications. *Clin. Exp. Metastasis* **24(8),** 599–608.
4. Porter, T., Stoddart, G., and Hinchliffe, A. (1998) The role of the plain X-ray in the assessment of prostatic symptoms. *Br. J. Urol.* **81(2),** 257–258.

**This article should be cited as follows:**

Chedgy, E.C.P., Niematallah, I., and Hawary, A. (2010) Osteosclerotic prostatic metastasis. *TheScientificWorldJOURNAL* **10**, 1330–1331. DOI 10.1100/tsw.2010.105.
